# Tuberculous Skull Base Osteomyelitis With Cerebral Venous Sinus Thrombosis in an Immunocompetent Adolescent: A Case Report

**DOI:** 10.7759/cureus.23865

**Published:** 2022-04-06

**Authors:** Rohini R, Prashant Badole, Saroj K Pati, Jhasaketan Meher, Nandhita Venkat

**Affiliations:** 1 Medicine, All India Institute of Medical Sciences, Raipur, IND; 2 Emergency Medicine, All India Institute of Medical Sciences, Raipur, IND; 3 Radiodiagnosis, All India Institute of Medical Sciences, Raipur, IND; 4 General Medicine, All India Institute of Medical Sciences, Raipur, IND

**Keywords:** young, thrombosis, tuberculosis, meningitis, skull-base osteomyelitis

## Abstract

Skull-base osteomyelitis is a rare yet lethal entity. It is infrequently observed among immunocompetent children and young adults, and Mycobacterium is much less common among the various bacterial and fungal etiological causes noted. We report a rare case of a 17-year immunocompetent girl who presented with complaints of head and neck pain and restricted neck movements. The analysis of her cerebrospinal fluid revealed a lymphocytic pleocytosis with elevated protein levels. Imaging studies revealed erosion of the occipital condyle and clivus and an extradural collection extending into the prevertebral and paravertebral spaces until the second cervical vertebra level. In addition to this life-threatening complication, the potential involvement of the cerebral venous sinuses is also of particular interest-a diagnosis of tubercular meningitis with skull base osteomyelitis based on the CSF and imaging findings. The drastic improvement in the initiation of anti-tubercular therapy emphasizes the need for prompt and early initiation of anti-tubercular therapy in endemic areas. The clinical picture, diagnosis, and treatment of tubercular skull-base osteomyelitis are further discussed, and pertinent literature has been reviewed.

## Introduction

Osteomyelitis is an inflammatory process associated with infection-induced bone destruction. Osteomyelitis is most common in the long bones like the tibia. Cranial osteomyelitis is rare and can affect the vault or skull base [1]. Retrospective studies have shown that 2% of all cases involve the skull [2]. Skull base osteomyelitis(SBO) is a rare and often fatal clinical entity. It usually occurs secondary to otogenic, sinogenic, odontogenic, or rhinogenic infections [3].

SBO is often misdiagnosed as malignancy [1]. It can be classified as typical when it involves the temporal bone and atypical occipital and sphenoid bones [4]. Both types can present with non-specific symptoms of fever and headache and progress to cranial neuropathies, meningitis, and venous thrombosis [4]. The most common causes of cranial osteomyelitis in developing countries are paranasal sinusitis, direct head injuries, and scalp infections [5]. The causative agents are usually bacteria and fungi. As a cause of SBO, Tuberculosis is extremely rare and has only been reported a few times [3,4,6,7]. We report a case of atypical SBO in an immunocompetent adolescent. It emphasizes the need for early diagnosis and prompt treatment, which led to a complete recovery with no neurological sequelae in our patient. 

## Case presentation

A 17-year old female with no known comorbidities presented to the Emergency Department of our hospital with insidious onset, progressive pain in the right side of the neck with restriction of neck movements for 2-3 months. It was followed by low-grade fever and headache for 15 days and depressed sensorium for one day. She had no similar complaints in the past. She had no history of contact with any patient with tuberculosis and no family history of neurological disease.

The patient had stable vitals on examination, but she was drowsy and irritable. Neck stiffness was present, and pupils were bilaterally sluggish in reaction to light. Oral cavity examination showed poor oral hygiene with a heavily coated tongue and grade 2 tonsillar hypertrophy. She had no history suggestive of a nasal or ear discharge. A provisional diagnosis of chronic meningitis secondary to possible mastoid/parapharyngeal pathology was made.

The patient’s complete blood picture showed raised absolute neutrophil count with an unremarkable picture. Erythrocyte Sedimentation Rate(ESR) was high at 55 mm in 1 hour. Blood culture showed no growth of bacteria after 48 hours. A guarded lumbar puncture under Mannitol cover was planned after CT (Computed Tomography). A cerebrospinal fluid(CSF) examination showed a leukocyte count of 200 cells/mm3 with 90% lymphocyte predominance and protein of 206.84 mg/dL Protein, 13 mg/dL glucose. CSF adenine deaminase(ADA) and lactate dehydrogenase(LDH) were normal. Staining for acid-fast bacilli (AFB) and cartilage-based nucleic acid amplification test (CBNAAT) for Mycobacterium tuberculosis in the CSF were negative.

**Figure 1:**
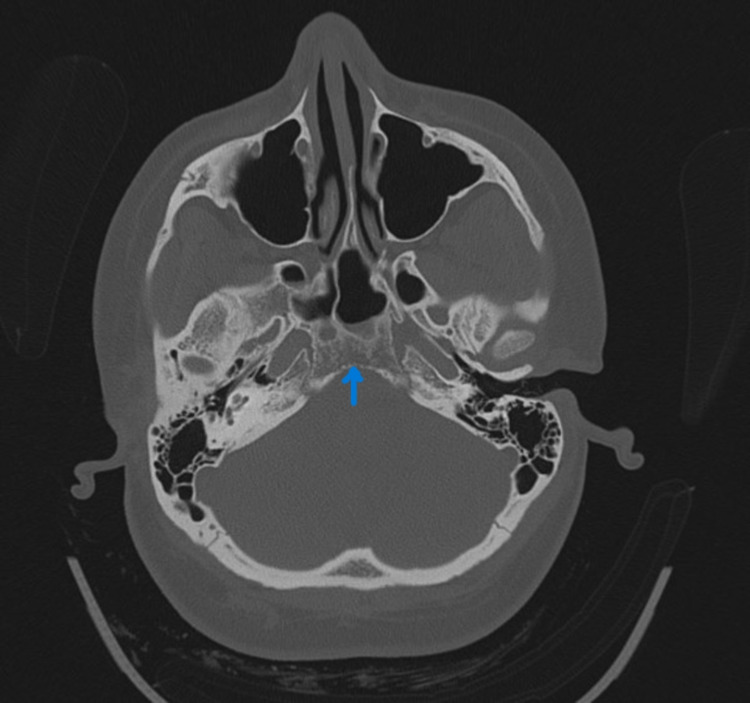
Axial CT at the skull base reveals erosion of basisphenoid bone.

**Figure 2:**
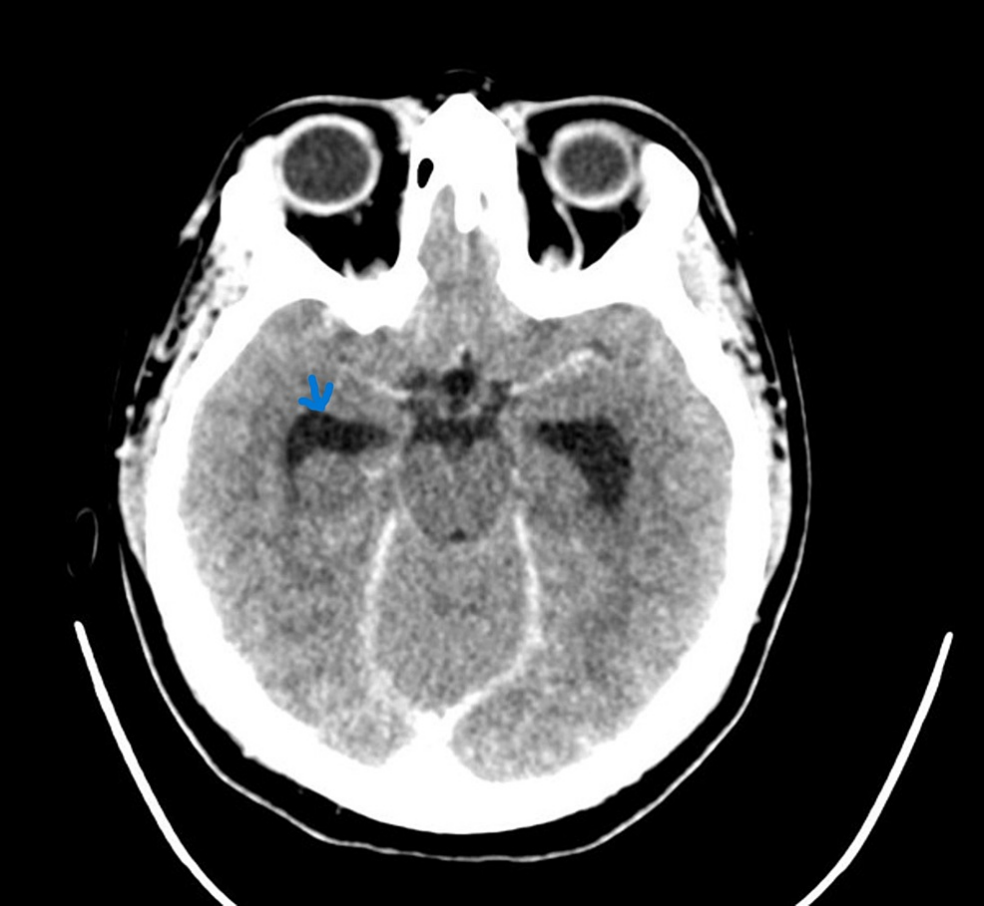
Post-contrast axial CT brain reveals dilated bilateral lateral ventricles with meningeal enhancement.

**Figure 3:**
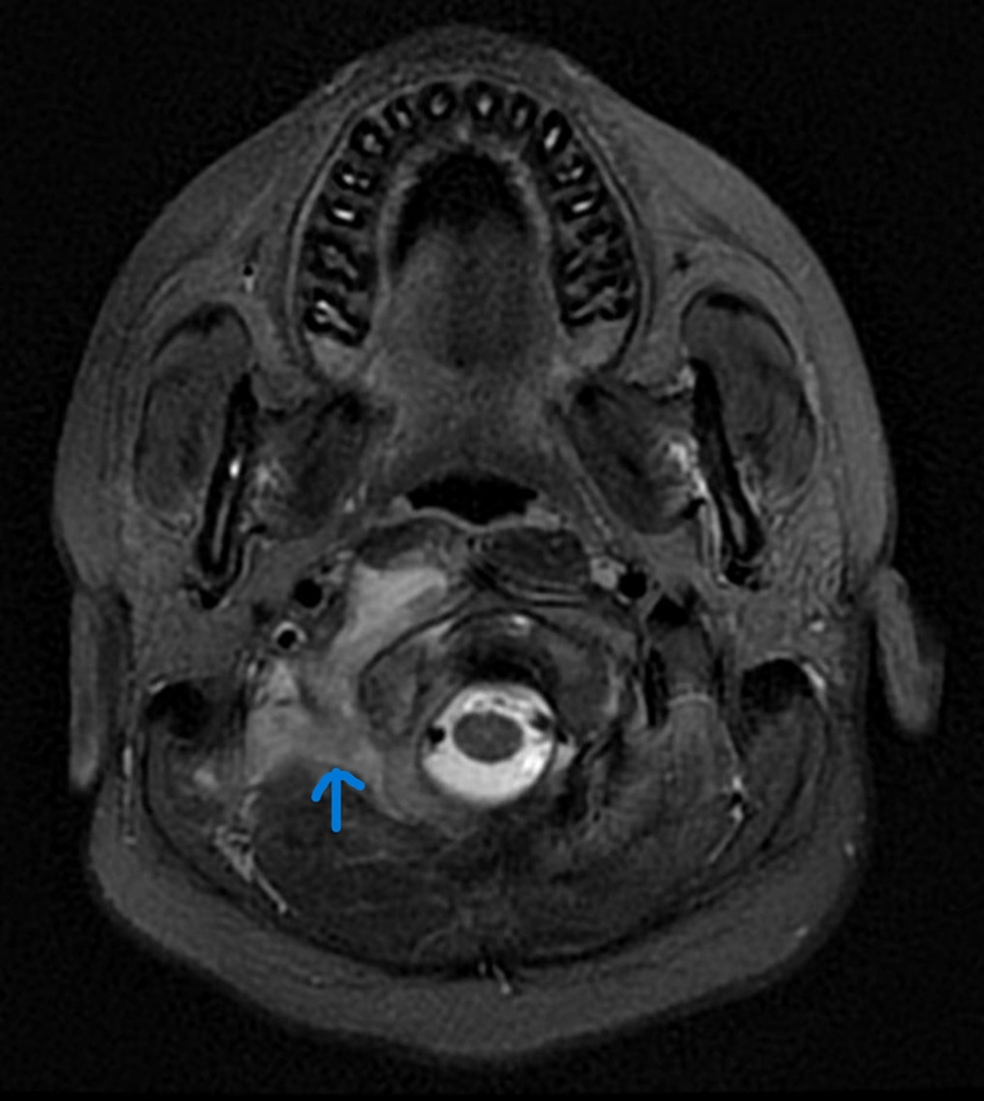
Axial MR STIR sequence reveals heterogeneously increased signal intensities in the right paravertebral space extending to the prevertebral space at the skull base.

**Figure 4:**
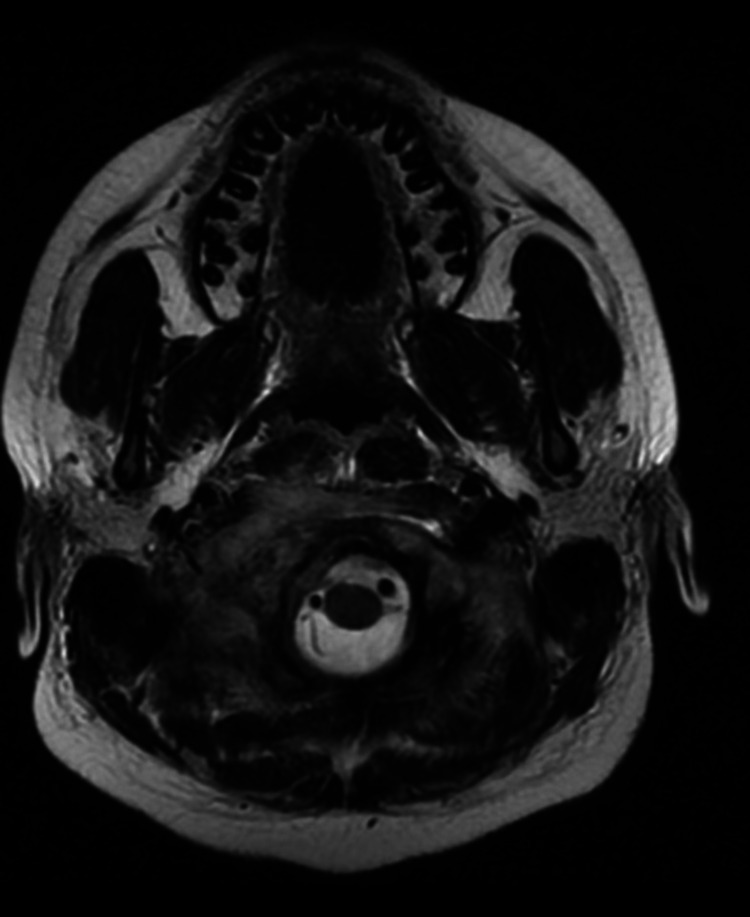
Axial MR T2 wt scan reveals intermediate to increased signal intensities in the right cerebellomedullary cistern & pre medullary cisterns.

**Figure 5:**
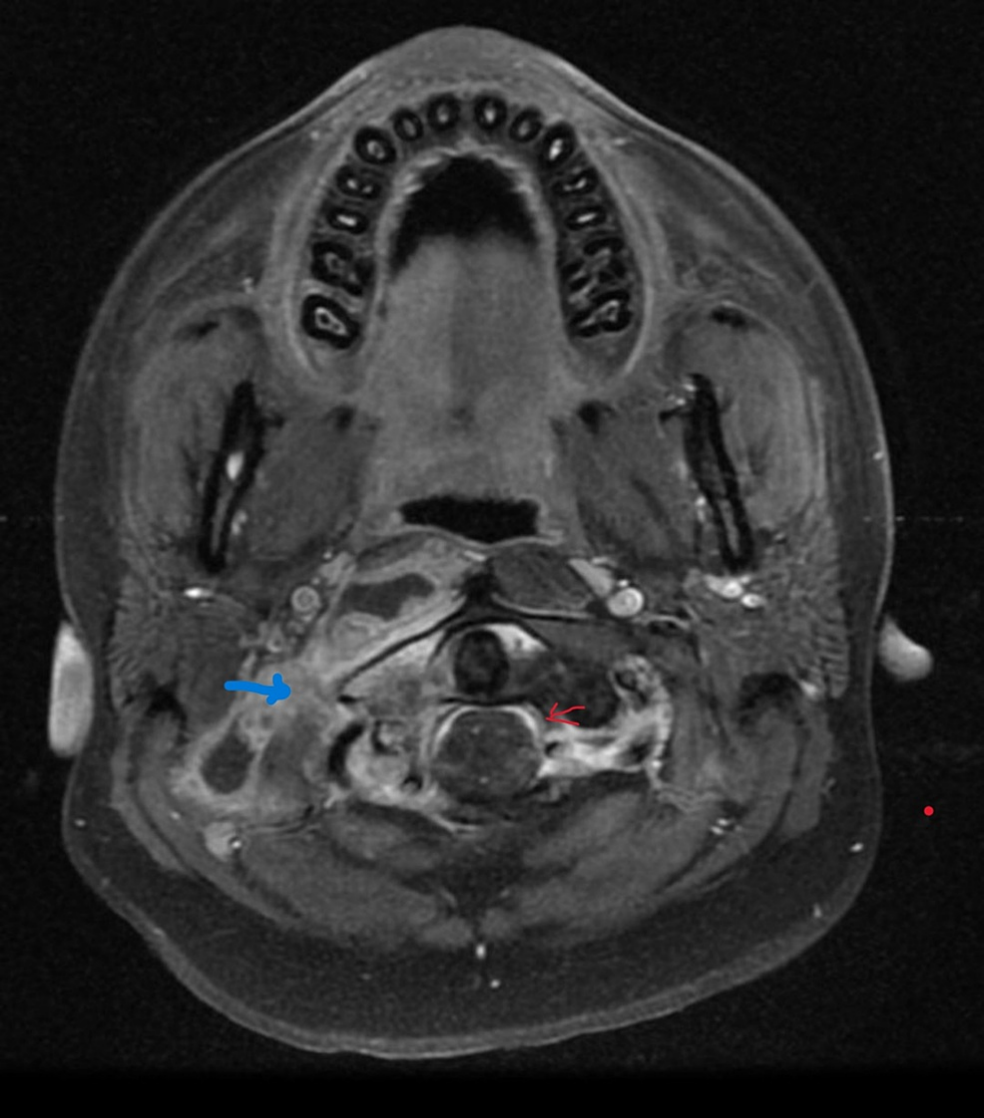
Axial T1 postcontrast sequence reveals thick, an irregular peripheral rim of enhancement in the right paravertebral region of the base of the skull and enhancing adjacent bone marrow of atlas vertebra representing skull base osteomyelitis with abscess formation (blue arrow). A note is made of enhancing meninges in the thecal space representing meningitis (red arrow).

**Figure 6:**
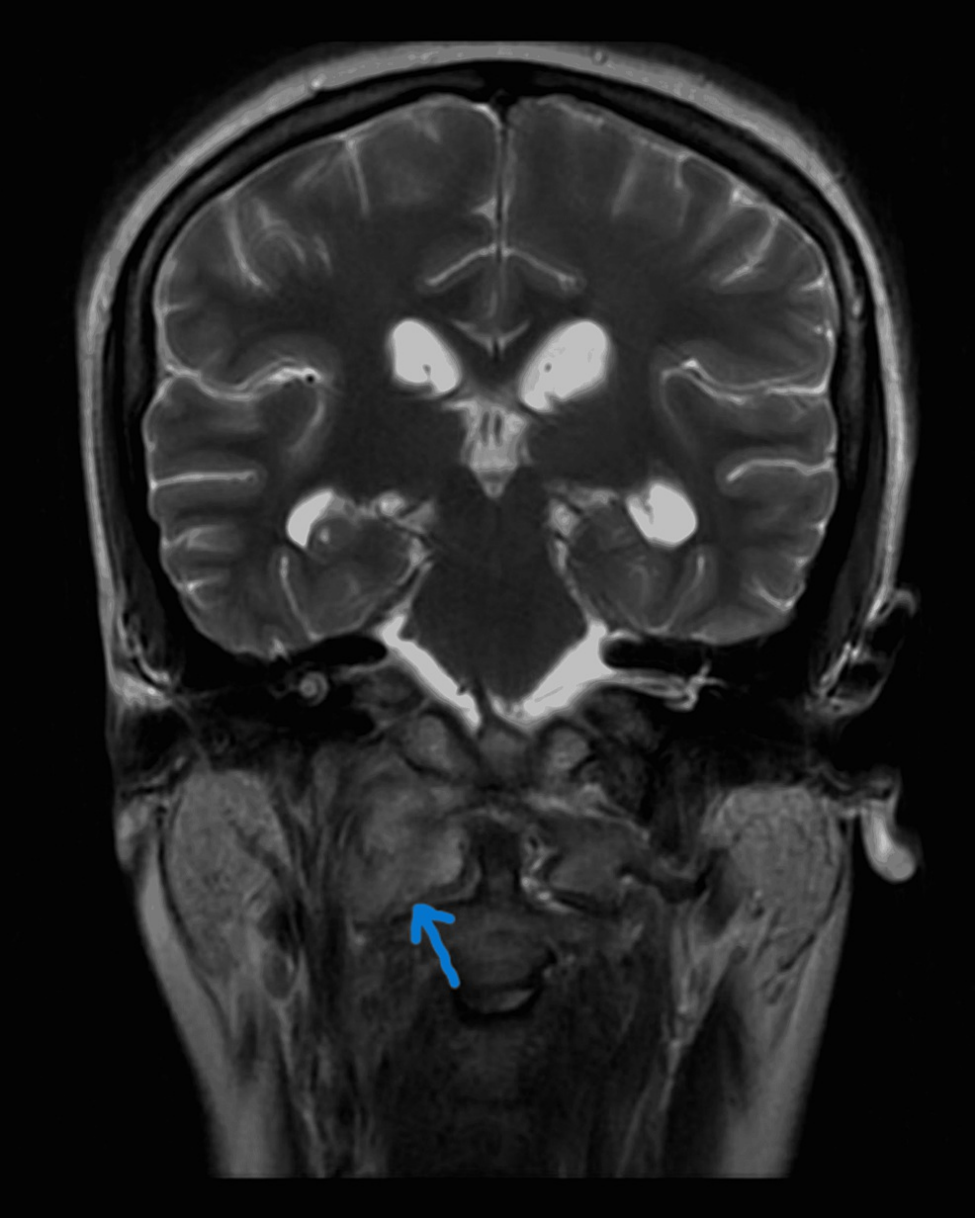
Coronal T2 scan reveals increased signal in right paraspinal space along C2 extending into adjacent skull base.

**Figure 7:**
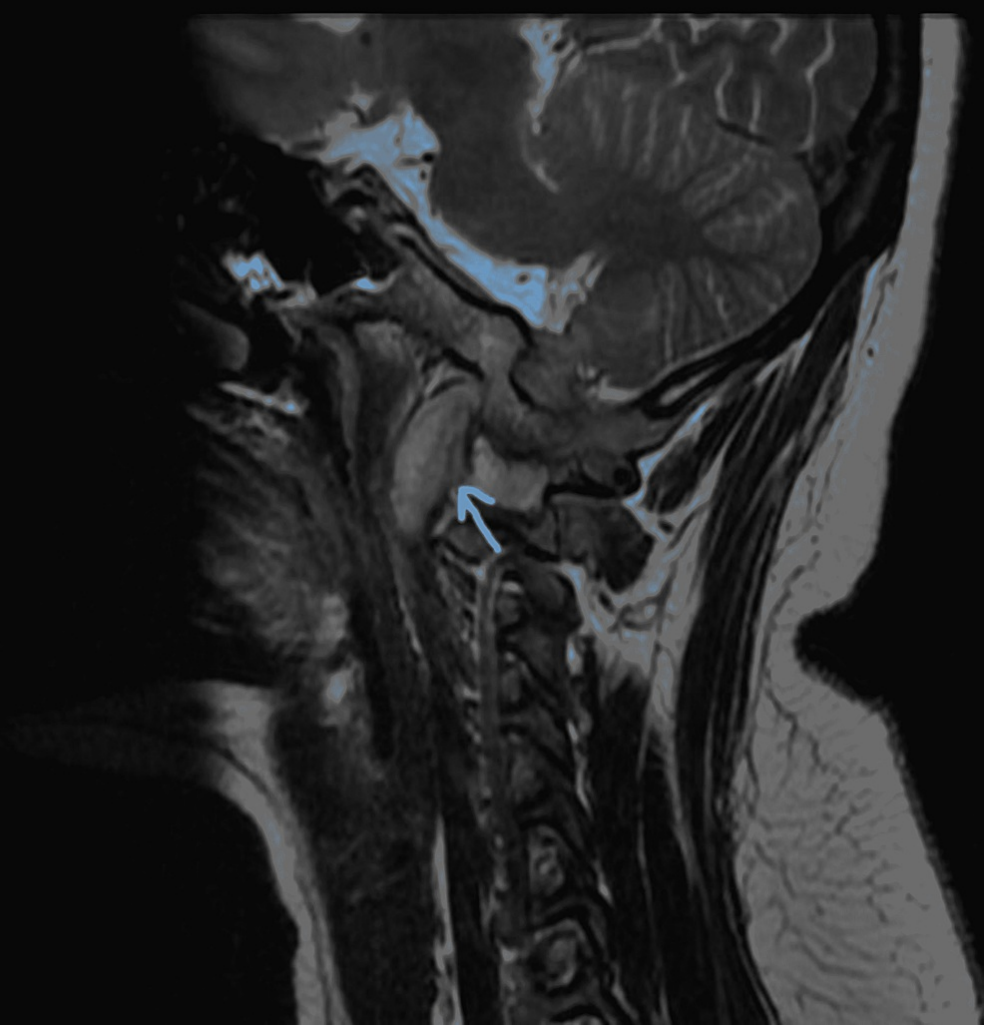
Right parasagittal T2 scan reveals the same as above.

**Figure 8:**
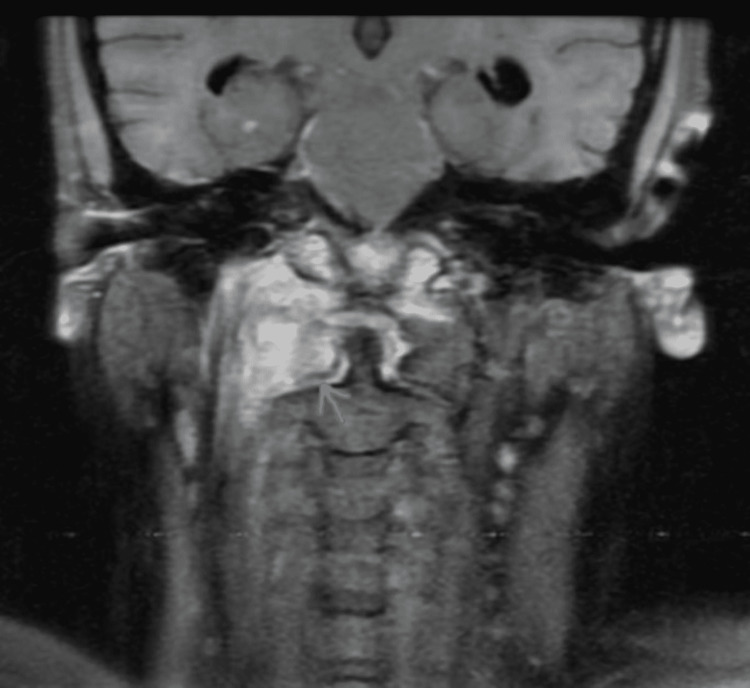
Coronal T1 postcontrast FS sequence shows heterogeneous enhancement.

**Figure 9:**
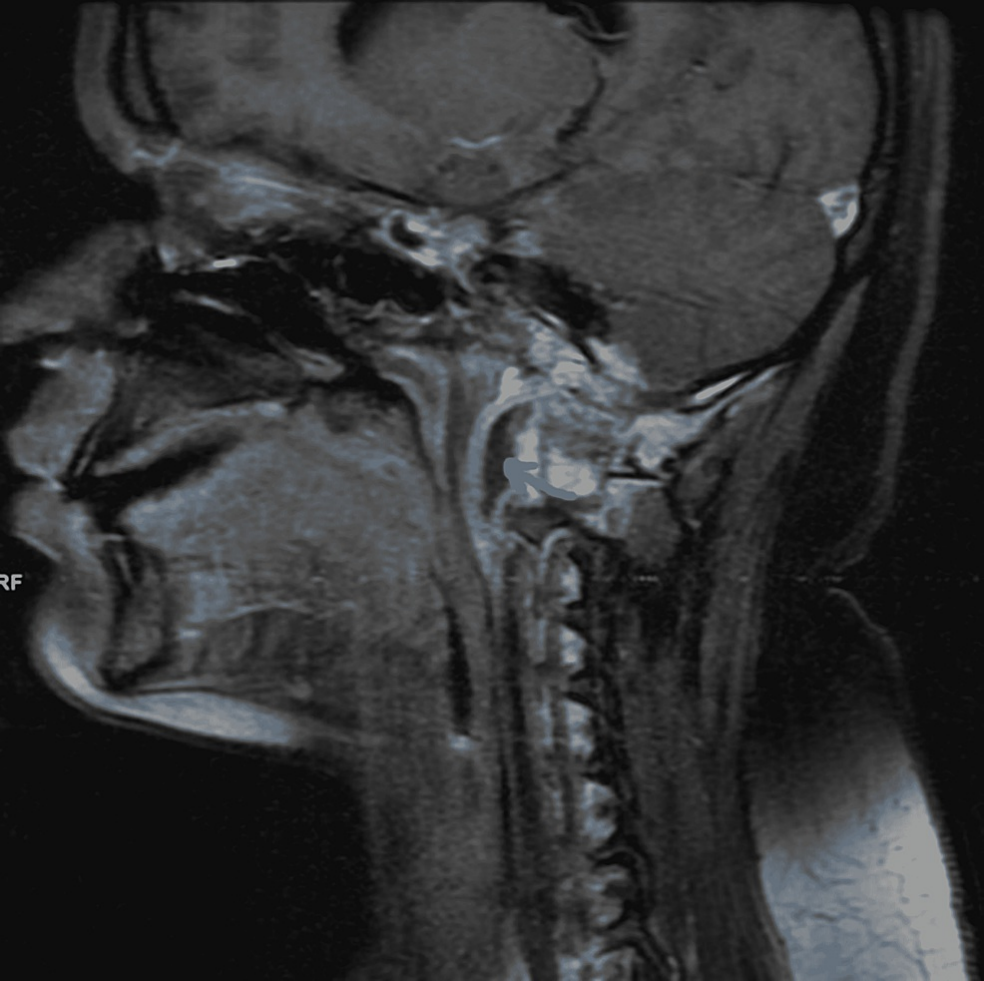
Right Parasagittal postcontrast T1 FS shows central non-enhancing area s/o necrosis.

CECT (Contrast-enhanced computed tomography) of the neck and temporal bone revealed bony erosion of the right occipital condyle, clivus, and basisphenoid (Fig 1) with cortical irregularity and loss of joint space. Thrombosis of distal transverse sinus extending to the right sigmoid sinus. Heterogeneously enhancing soft tissue attenuation contents with few small liquefaction areas in prevertebral space to the right of midline, extending from clivus to C2 vertebra, was noted along with communicating hydrocephalus (Fig 2). MRI revealed a prevertebral, paravertebral, extradural peripheral-enhancing collection showing internal communication with each other suggestive of possible skull-base osteomyelitis (Fig 3, 4). MRI study also revealed abscess formation with meningitis (Fig 5). Figures 6-9 show coronal and sagittal views of the same findings. The imaging revealed no abnormalities within the paranasal sinuses or the external auditory canal and middle ear. An attempt was made for CT-guided aspiration of the collection, but it was not possible due to poor liquefaction.

Based on these reports, the patient was diagnosed with a case of atypical skull-base osteomyelitis with meningitis with cerebral venous sinus thrombosis secondary to tuberculosis. She was started on weight-based four-drug anti-tuberculosis treatment (ATT) containing rifampicin, isoniazid, pyrazinamide, ethambutol, and dexamethasone. She was also started on anticoagulation with a vitamin K antagonist. Blood and CSF cultures were sterile, showing no growth.

**Figure 10:**
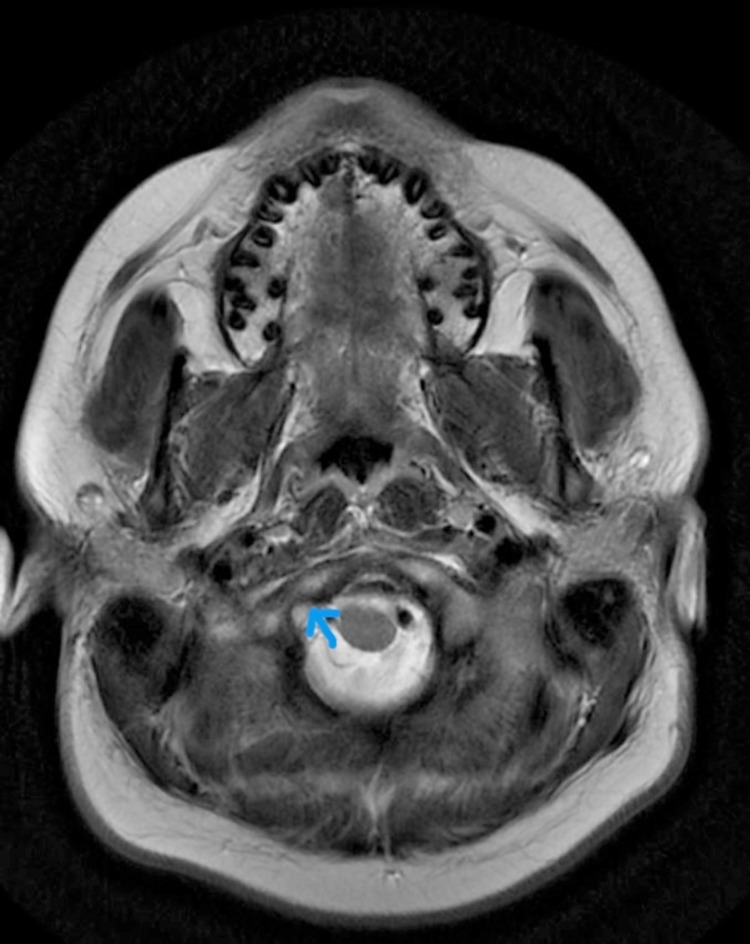
T2W axial section shows resolution of altered signal

**Figure 11:**
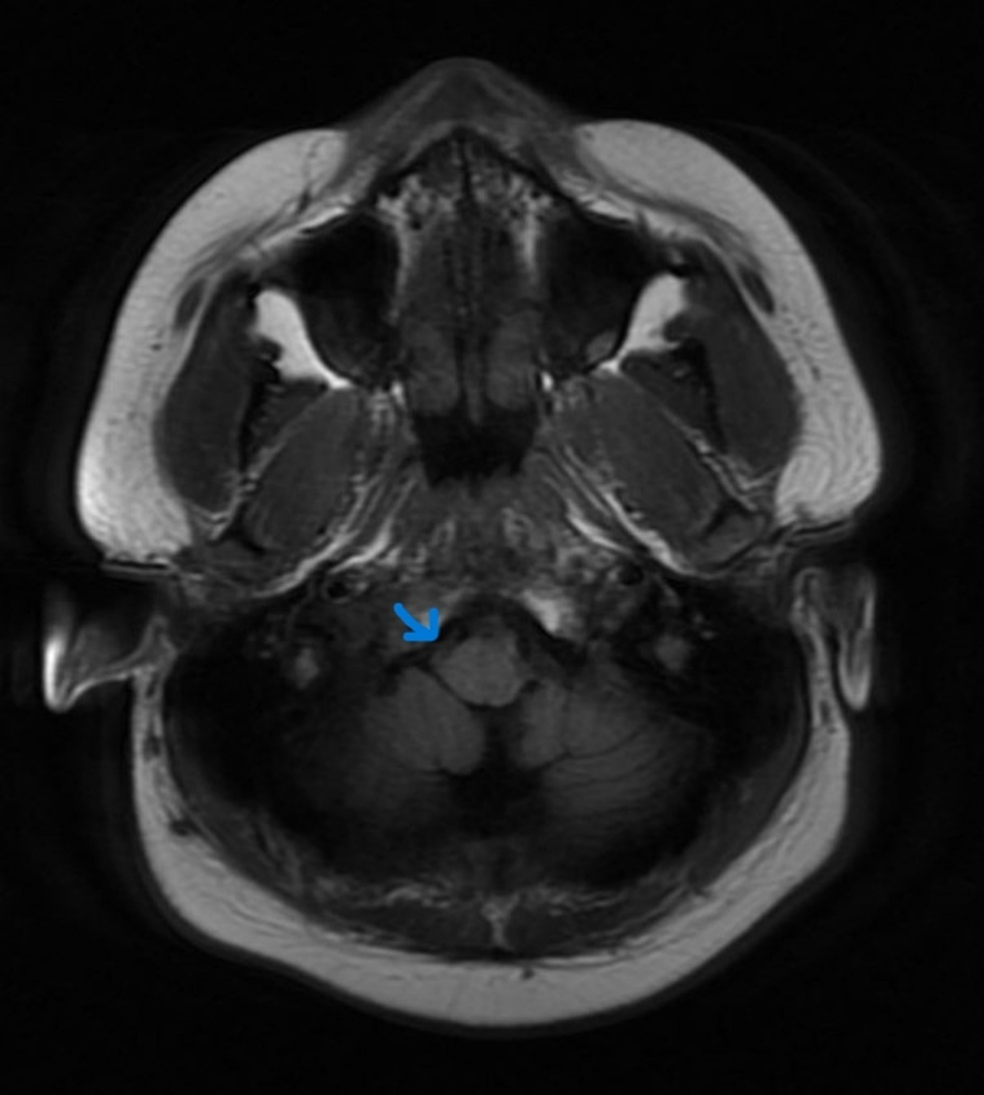
T1W axial scan reveals resolution of altered signal

Despite the initiation of ATT, the patient continued to develop a fever for a month. When she was re-evaluated, her radiological investigation showed partial resolution of the prevertebral, paravertebral, and extradural collection (Fig 10, 11). The patient became afebrile and asymptomatic, with a return of regular movement and activity after six weeks of initiation of ATT. The patient has currently completed nine months of ATT. She is asymptomatic and doing well.

## Discussion

Skull-base osteomyelitis (SBO) occurs more commonly in immunosuppressed patients with immunodeficiency syndromes or diabetes mellitus, and the risk generally increases with age [8]. In recent literature, SBO has been classified as typical and atypical based on the site and mode of involvement. Typical SBO has been described as a complication of otogenic, odontogenic, or sinus infection involving the temporal and occipital bones [4]. The most common organisms causing malignant otitis externa (MOE) are Pseudomonas aeruginosa and Staphylococcus aureus, which may cause typical SBO [9]. 

Atypical skull-base osteomyelitis occurs much less frequently, and unlike typical SBO, it is more often seen following a sinus infection over an ear infection. More associated with the sphenoid and occipital bones, it may only present with headaches [4]. It has been observed in children, both immunocompetent and immunodeficient (anemia, malnutrition, diabetes, leukopenia) [10]. A case study series from 1946 to 2013 revealed 42 cases of central/atypical SBO unrelated to ear infection, with a mean age of 52 years and a male-to-female ratio of 2.2:1 [10]. In contrast, our case is a 17-year-old female. Table 1 reviews cases of atypical SBO reported in the past 10 years, showing their clinical presentations and outcomes.

**Table 1:** A review of all cases of atypical SBO in past 10 years.

Sl. No.	Article	Year of publication	Clinical presentation	Organism isolated	Treatment given	Outcome
1.	Iyer AS, Patil PV, Pandey D, Kute BS. Tubercular skull base osteomyelitis - A case report. cases. [3]	2022	Stroke in a 12-year-old immunocompetent girl	Mycobacterium tuberculosis	Surgical debridement with antitubercular treatment	Recovered
2.	Fu, Ze-Ming MD; Zhang, De-Jun MD; Guan, Guo-Fang MD A Case of Atypical Skull Base Osteomyelitis Secondary to Otitis Media Due to Delayed Diagnosis, Journal of Craniofacial Surgery [11]	2021	Atypical SBO secondary to otitis media in 84yrs old diabetic male	Methicillin-resistant Staphylococcus aureus	Surgical debridement with >8weeks of vancomycin therapy	Improved
3.	Sanjay Kumar, Ashok Kumar, Hrishikesh Gadhavi, Vikas Maheshwari, Tubercular skull base osteomyelitis in an immunocompetent individual: A rare entity“, Interdisciplinary Neurosurgery [4]	2021	Sphenoid sinusitis leading to atypical SBO in a 34 year old male	Mycobacterium tuberculosis	ATT	Recovered
5.	Suma Radhakrishnan, Hiba Mujeeb, Chandni Radhakrishnan, Central skull base osteomyelitis secondary to invasive aspergillus sphenoid sinusitis presenting with isolated 12th nerve palsy, IDCases, [12]	2020	Unilateral lower motor neuron type 12^th^ cranial nerve palsy with atypical SBO in a diabetic patient on immunosuppressants for myasthenia gravis	Aspergillus	Endoscopic debridement with 10 weeks course of Voriconazole	Improved
6.	See A, Tan TY, Gan EC. Atypical culture-negative skull base osteomyelitis masquerading as advanced nasopharyngeal carcinoma. Am J Otolaryngol [13]	2016	Unilateral 3^rd^, 4^th^, 6^th^, 9^th^, 10^th^ and 12^th^ cranial nerves palsy with a nasopharyngeal mass masquerading as a malignancy in a 54 year old man with diabetes and hypertension	Culture negative	Endoscopic debridement with 6 weeks course of broad spectrum antibiotics	Partial recovery
7.	Lee SJ, Weon YC, Cha HJ, Kim SY, Seo KW, Jegal Y, Ahn JJ, Ra SW. A case of atypical skull base osteomyelitis with septic pulmonary embolism. J Korean Med Sci [14]	2011	Unilateral 9^th^, 10^th^ and 12^th^ cranial nerve palsy with mastoiditis, venous thrombosis in the transverse and sigmoid sinuses, thrombophlebitis and SBO with multiple peripheral pulmonary nodules of varying degrees of cavitation in both lungs as a result of septic emboli in a 51 year old male	Enterobacter aerogenes	Systemic antibiotics for 8 weeks with anticoagulation for 1 year	Recovered

In a retrospective study done by Urvashi Singh et al. in Chennai, India, they found only 10 documented cases of atypical SBO. Of these 10 patients, eight were diabetic; all had some cranial nerve involvement, and one was diagnosed with extrapulmonary tuberculosis based on clinical suspicion alone [15].

Among bacterial causes, studies state that SBO commonly occurs due to Pseudomonas aeruginosa infection, while fungal infections are seen more with Aspergillus, Candida spp., and mucormycosis [2]. However, reports of SBO secondary to mycobacterial causes are very scant. In regions such as India, where the burden of tubercular disease is high and compounded by high rates of post-primary dissemination among young adults and pediatric groups, CNS TB manifests more commonly as tuberculous meningitis intracranial tuberculoma and spinal tuberculous arachnoiditis. Table 2 shows all the reported cases of tuberculosis leading to SBO. It can be noted that all the cases were reported from India.

**Table 2:** Reported cases of Tubercular SBO

Sl. No.	Article	Year of publication	Clinical presentation	Diagnosed by	Outcome
1.	Bhavanam HS, Rajesh A, Uppin MS: Tubercular osteomyelitis of spheno-clival region presenting with lateral rectus palsy. Neurol India [6]	2014	Fever, unilateral 6^th^ cranial nerve palsy in a 20-year-old male. Imaging suggestive of sphenoid sinus mass with the destruction of clivus	Granulomas on biopsy with Mantoux positive	Recovered
2.	Sagar P: Tubercular osteomyelitis of the clivus. Turk j ear nose throat. [7]	2018	Headache, diplopia. Imaging suggestive of nasopharyngeal mass with erosion of clivus, obstructive hydrocephalus, and infarction of basal ganglia and thalamus	Biopsy findings	Died
3.	Sanjay Kumar, Ashok Kumar, Hrishikesh Gadhavi, Vikas Maheshwari, Tubercular skull base osteomyelitis in an immunocompetent individual: A rare entity“, Interdisciplinary Neurosurgery [4]	2021	Sphenoid sinusitis leading to atypical SBO in a 34-year-old male	Biopsy showing inflammatory granuloma	Recovered
4.	Iyer AS, Patil PV, Pandey D, Kute BS. Tubercular skull base osteomyelitis - A case report. cases [3]	2022	Stroke in a 12-year-old immunocompetent girl. Imaging revealed erosion of anterior and posterior clinoid processes and occipital protuberance with vasculitic infarct in the pons	GeneXpert positive in the biopsy sample and CSF findings	Recovered
5.	Our case		Headache and features of meningitis in a 17 year old immunocompetent girl. Imaging revealed atypical SBO with meningitis, hydrocephalus, and para- and prevertebral collections and dural sinus thrombosis	CSF findings	Recovered

SBO commonly presents as ear pain and discharge, nasal stuffiness and discharge, facial and periorbital puffiness, hearing loss, difficulty in swallowing, and cranial nerve palsies. Complications that occur secondary to skull-base osteomyelitis include labyrinthitis, intracranial abscess, retropharyngeal abscess, venous sinus thrombosis, carotid artery involvement, and neuropathic cranial nerves. Sinovenous thrombosis is a complication of SBO that most commonly affects the transverse-sigmoid sinus (typical), cavernous sinus (atypical), and internal jugular vein (both) [2]. Thrombus formation occurs secondary to continued inflammatory responses and can spread and grow into other venous sinuses and cause subsequent occlusion and embolism. The constellation of complications that occur secondary to SBO, such as sinovenous thrombosis, meningitis, abscesses, and ischemic infarcts, is called Lemierre syndrome. LS is associated with a 4-12% [16] and requires a prolonged course of antibiotic treatment (8-12 weeks).

Computed Tomography or CT imaging might even be normal in very early stages. In cranial neuropathy, imaging of the skull is best obtained with MRI due to better soft tissue discrimination and assessment of planes around the skull base [17]. Characteristic Magnetic-Resonance (MR) findings include diffuse clival hypointensity on T1-weighted images secondary to bone marrow infiltration [18]. CT is a poor choice for SBO to represent an intracranial extension, bone marrow involvement, and measure treatment response. Bone SPECT (single photon emission CT) scintigraphy and MRI are superior to CT scans for detecting early SBO. MRI is also more sensitive in determining the extent of the disease. In-111 WBC bone scintigraphy is helpful for the anatomic localization of lesions, especially in the skull base, and can be used to assess for active SBO after treatment [2]

Clinical and imaging differential diagnoses of SBO include neoplastic causes (squamous cell carcinoma of the head and neck, lymphoma, hematogenous metastasis involving the clivus, leukemia) and non-neoplastic processes (inflammatory pseudotumor, Wegener’s granulomatosis, tuberculosis, sarcoidosis, fibrous dysplasia, Paget’s disease) [17].

Treatment of osteomyelitis includes antibiotic courses as the mainstay of treatment. However, empirical therapies are initiated until culture-driven antibiotic sensitivity reports are obtained. These might lead to partially treated infection and potentially increase the degree of antibiotic resistance. For culture-positive SBO, antibiotics as per sensitivity reporting should be administered. Hyperbaric oxygen has also been suggested in refractive cases [19]. Surgery is usually indicated for debridement and helps identify the causative organism, as noted in Table 1,2. In our case, surgery was deferred by the neurosurgeon as the collection was not well liquified, and the patient was showing signs of improvement with medical management alone.

## Conclusions

Despite cultures and CBNAAT for mycobacterium tuberculosis being negative, ATT was initiated in this patient, which showed drastic clinical improvement. This report emphasizes the need for prompt and early initiation of anti-tubercular therapy when there is a high degree of clinical suspicion, particularly in areas of high case load, such as India. This is the first report on atypical, tubercular skull-base meningitis with cerebral venous sinus thrombosis in a previously healthy adolescent.
